# PPARα modulates gene expression profiles of mitochondrial energy metabolism in oral tumorigenesis

**DOI:** 10.7603/s40681-016-0003-7

**Published:** 2016-02-22

**Authors:** Yi-Ping Huang, Nai Wen Chang

**Affiliations:** 1 Department of Physiology, College of Medicine, China Medical University, 404 Taichung, Taiwan; 2 Department of Biochemistry, College of Medicine,, China Medical University, 404 No. 91, Hsueh-Shih Road, Taichung, Taiwan

**Keywords:** Fenofibrate, Oral cancer, PPARα, RT^2^ profiler PCR array

## Abstract

Metabolic reprogramming plays a crucial role in the development of cancer. The aim of this study was to explore the effect of fenofibrate, an agonist of peroxisome proliferator-activated receptor alpha (PPARα), on gene expression profiles of mitochondrial energy metabolism. Our results showed that PPARα expression was negatively correlated with tumor progression in an oral cancer mouse model. Activation of PPARα through fenofibrate suppressed migration of oral cancer cells. Differential protein profiling demonstrated that expressions of genes related to mitochondrial energy metabolism were either up-regulated (*Atp5g3, Cyc1, Ndufa5, Ndufa10*, and *Sdhd*) or down-regulated (*Cox5b, Ndufa1, Ndufb7*, and *Uqcrh*) through PPARα activation and response. Our results indicate that PPARα exhibits a great potential for anti-oral cancer therapies by modulating cancer cell mitochondrial energy metabolism.

## 1. Introduction

One of the prominent characteristics of rapidly growing cancer cells is their capacity to sustain high rates of glycolysis for ATP production regardless of whether oxygen is present—a phenomenon known as the Warburg effect [1]. Activators of peroxisome proliferator-activated receptors (PPARs) have been shown to exhibit a great potential for anticancer therapies by modulating cancer cell energy metabolism and signaling pathways [2, [3]. PPARα is known to modulate the expression of genes regulating glucose and lipid metabolism [4, [5]. Fenofibrate is a synthetic agonist of PPARα and a widely used hypolipidemic drug with antiinflammatory and anti-atherosclerotic effects in humans [6, [7]. Fenofibrate has been reported to be involved in several anticancer activities, including induction of apoptosis, reduction of the proliferation rate, attenuation of IGF-1 receptor signaling, inhibition of tumor angiogenesis, and suppression of the inflammatory response and oxidative stress in cancer cells like melanoma, mantle cell lymphoma, medulloblastoma, glioma, and endometrial cancer cells [8-[14]. However, the anticancer activity of fenofibrate in energy homeostasis is not well clarified or understood.

Our previous studies suggest a beneficial role of fenofibrate in anti-oral tumorigenesis in both cell culture and animal models. Fenofibrate inhibits the invasion and migration of CAL 27 oral cancer cells by suppressing the protein expressions of matrix metalloproteinase-1 (MMP-1), MMP-2, MMP-7, and MMP-9 through the AMPK and NF-κB signaling pathway [15]. Fenofibrate causes the reduction in the incidence and size of squamous carcinoma and suppresses the progression of the preneoplastic lesion into squamous cell carcinoma in an oral-specific 4-nitroquinoline 1-oxide/arecoline mouse model [16]. Recently, we demonstrated that fenofibrate provided novel mechanisms for delaying oral tumor development via the reprogramming of metabolic processes by interrupting the binding of hexokinase II to the voltage-dependent anion channel and increasing metabolites in the tricarboxylic acid cycle (unpublished data). It is possible that part of the anticancer mechanisms of fenofibrate might involve regulating the gene expression of mitochondrial energy metabolism. Therefore, this study focused on the changes in genes expression of mitochondrial energy metabolism in oral cancer cells treated with fenofibrate. The protein expression levels of PPARα were also examined in oral tumor progression.

## 2. Materials and methods

### 2.1. Administration of 4-NQO and arecoline

Seventy 6-week-old male C57BL/6JNarl mice were purchased from the National Laboratory Animal Center. The mice were handled in accordance with the Animal Care and Use Guidelines of the China Medical University, and the study protocol was approved by the Institutional Animal Care Use Committee. The experiments were controlled as previously described [17]. The carcinogens, 200 μg/ml 4-nitroquinoline 1-oxide (4-NQO; Sigma- Aldrich, St. Louis, MO, USA) and 500 μg/ml arecoline hydrobromide (Tokyo Chemical Industry Co. LTD, Tokyo, Japan), were used to induce oral tumorigenesis in the mice for a period of 8 weeks and then observed for the indicated time including 0, 8, 12, 16, 20, 24, and 28 weeks. Ten mice were euthanized at each indicated time. During the administration, all mice were allowed to access the drinking water and chow diet (Prolab® RMH 2500 PMI Nutrition International, LLC, MO, USA) ad libitum. The experiments were carried out under controlled conditions with a 12-h light/dark cycle.

### 2.2. Cell culture and wound healing assay

The 28-week mouse oral cancer cells were obtained from primary cultured cells of tongue cancer induced by 4-NQO (200 μg/ml) and arecoline (500 μg/ml) [16]. Cells were grown in DMEM supplemented with 10% fetal bovine serum, 1% antibiotic-antimycotic and 2 mM L-glutamine (Gibco; Life Technology Corporation, NY, USA) at 37°C in a humidified 5% CO_2_ atmosphere incubator. For migration assay, 5 × 10^4^ cells were seeded in ibidi Culture-Insert (ibidi GmbH, Martinsried, Germany) on a 24 well plate. After appropriate cell attachment, the Culture-Insert was gently removed, and cells were then incubated with or without fenofibrate (50 μM). Next cells were allowed to migrate into the wound area for 0, 16, 24, and 38 h. Cells in the wound area were photographed and measured at an image analysis platform. (http://ibidi.com/applications/wound-healing-and-migration/)

### 2.3. RNA Extraction and RT^2^ Profiler PCR Array examination

The 28-week mouse oral cancer cells were incubated with 50 μM fenofibrate for 18 h, and an equivalent volume of 0.1% DMSO was used as control. Total RNA was extracted from the cells with or without fenofibrate treatment by using the Trizol reagent solution (Ambion, Life Techniologies) according to the manufacturer’s recommendations. The quality of RNA samples was determined by Agilent 2100 Bioanalyzer analysis using a RNA 6000 Nano Kit (Agilent Technologies, Inc.). The quantity of RNA samples was determined using a NanoDrop ND-1000 spectrophotometer (Thermo Scientific). c-DNA was converted using a RE3 Reverse Transcriptase Mix (Qiagen Ltd.). Quantitative PCR was performed according to the RT^2^ Profiler PCR array instructions on a 7300 Real-Time PCR System (Applied Biosystems, CA, USA). The Mouse Mitochondrial Energy Metabolism (PAMM-008Z) RT^2^ Profiler PCR Array, which profiles a total of 84 genes expression, was purchased from Qiagen Ltd. The PCR cycling condition was set as follows: 1 cycle of 95°C for 10 min; 40 cycles of 95°C for 15 sec and 60°C for 1 min; and 1 cycle of 95°C for 15 sec, 60°C for 30 sec, and 95°C for 15 sec. The results of Ct values were submitted to the Web-based PCR array data analysis software, which is available at www.SABioscience.com/pcrarraydataanalysis.php.

### 2.4. Protein extraction and Western blotting

To analyze PPARα protein levels in mouse tongue tissue, each sample at the indicated time was homogenized and proteins were extracted as previously described [18]. Total protein contents were determined using a Bio-Rad protein assay reagent (Bio- Rad Laboratories, CA, USA) with bovine serum albumin as a standard. Equal amounts (50 μg) of extracted proteins from each condition were fractionated by 12% SDS-Polyacryamide gels and transferred onto a polyvinylidene difluoride (PVDF) membrane (Millipore, MA, USA). The Western blots were probed with a PPARα primary antibody (Santa Cruz Bioechnology, TX, USA) and then incubated with horseradish peroxide-conjugated secondary antibodies. The immunoreactive protein bands were detected by a SuperSignal Enhanced Chemiluminescence (Millipore, MA, USA). Western blots were quantified by densitometric analysis using KODAK image analysis software (Kodak EDAS290, Eastman Kodak, Rochester, NY, USA).

**Figure 1:**
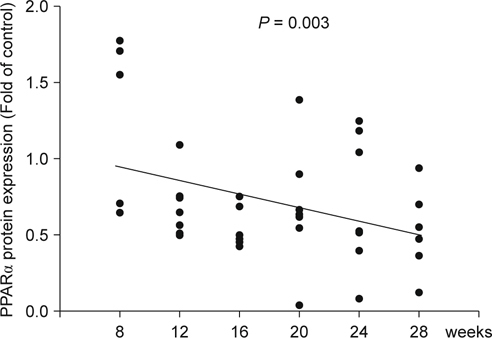

### 2.5. Statistical analysis

All data were analyzed using SPSS 15.0 for Windows. Statistical analyses of the PPARα expression were performed using Linear regression analysis. A *P*-value of less than 0.05 was considered statistically significant.

## 3. Results

### 3.1. PPARα protein expression levels in tongue tissues

First, we determined the PPARα protein levels of tongue tissues in an 4-NQO/arecoline induced mouse model. The PPARα protein levels at each time point are shown in Figure 1. The PPARα protein levels were progressively decreased in a time-dependent manner over a 28-week observation period when compared with the control (0 week). Linear regression analysis showed that PPARα expression was negatively correlated with the advancing of tumor development (*P* = 0.003).

### 3.2. Activation of PPARα suppressed migration of oral cancer cells

**Figure 2:**
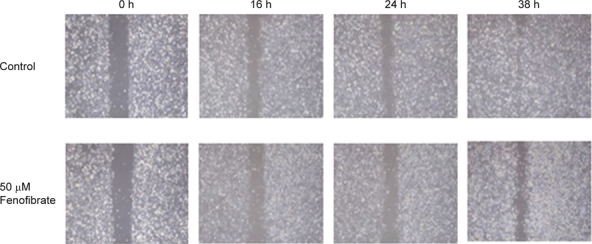

We next assessed whether activation of PPARα influenced cancer cell migration. The 28-week mouse oral cancer cells were treated with fenofibrate (0 or 50 μM) for 0, 16, 24, and 38 h. We found that fenofibrate decreased cell migration ability up to the observation time of 38 h (Figure 2).

**Table 1:** 

	Average Ct		Average ΔCt		2^-ΔCt^		Fold-change
Gene Symbol	Control	Fenofibrate	Control	Fenofibrate	Control	Fenofibrate	As Compared to control
*Up-regulating mRNA expression levels*							
*Atp5g3*	25.15	23.40	4.77	3.06	0.037	0.120	3.272
*Cyc1*	31.14	29.05	10.76	8.71	0.001	0.002	4.141
*Ndufa10*	29.78	27.79	9.40	7.45	0.001	0.006	3.864
*Ndufa5*	33.37	32.09	12.99	11.75	0.000	0.000	3.864
*Sdhd*	UD	32.48	14.62	12.14	0.000	0.000	5.579
*Down-regulating mRNA expression levels*							
*Cox5b*	32.17	UD	11.79	14.66	0.000	0.000	0.137
*Ndufa1*	31.71	UD	11.33	14.66	0.000	0.000	0.099
*Ndufb7*	33.05	UD	12.67	14.66	0.000	0.000	0.252
*Uqcrh*	19.04	20.26	-1.34	-0.08	2.532	1.057	0.418

UD: Undetermined. The Ct values greater than 35 show as UD. Fold-change values greater than one indicate a positive- or an up-regulation; however, the fold-change values less than one indicate a negative or down-regulation.

### 3.3. mRNA expression levels of genes involved in mitochondrial energy metabolism after fenofibrate treatment

To explore the expression levels of genes involved in mitochondrial energy metabolism through PPARα activation and response, oral cancer cells from the 28-week group were treated with or without 50 μM fenofibrate for 18 h. Table 1 shows the changes in the mRNA expression levels of 9 genes involved in mitochondrial energy metabolism detecting by an RT^2^ Profiler PCR Array. Fold-change (2^-ΔΔCt^) is measured as the level of normalized gene expression (2^-ΔCt^) in the fenofibrate-treated group divided by the level of normalized gene expression (2^-ΔCt^) in the control group. Fold-change values greater than one indicate a positive- or an upregulation; however, fold-change values less than one indicate a negative or down-regulation. The expression levels of *Atp5g3* (ATP synthase, H^+^ transporting, mitochondrial F0 complex, subunit C3), *Cyc1* (Cytochrome c-1), *Ndufa5* (NADH dehydrogenase (ubiquinone) 1 alpha subcomplex, 5), *Ndufa10*, and *Sdhd* (Succinate dehydrogenase complex, subunit D, integral membrane protein) genes were up-regulated in fenofibrate-treated cells compared with those in control cells. Conversely, expressions of *Cox5b* (Cytochrome c oxidase, subunit Vb), *Ndufa1*, *Ndufb7* (NADH dehydrogenase (ubiquinone) 1 beta subcomplex, 7), and *Uqcrh* (Ubiquinol-cytochrome c reductase hinge protein) genes were down-regulated. These findings indicate that PPARα activation and response might modulate mitochondrial function and energy production.

## 4. Discussion

It is well known that cancer cells undergo significant metabolic adaptations in energy metabolism [1]. Shifting the balance between the glycolytic and mitochondrial processes could be an important point in cancer therapy. In this study, we found that PPARα protein levels were negatively associated with tumor development in an oral cancer mouse model (Figure 1). Activation of PPARα by fenofibrate decreased the migration ability of oral cancer cells *in vitro* (Figure 2) and suppressed the tumor progression into squamous cell carcinoma *in vivo* [16]. We thought that these molecular events might be linked to interrupting the Warburg effect through reprogramming ATP production pathway. Therefore, the transcription levels of genes involved in mitochondrial energy metabolism were determined by an RT^2^ Profiler PCR Array. We found that nine genes’ transcription levels in the respiratory chain, including *Ndufa1, Ndufa5, Ndufa10, Ndufb7, Sdhd, Cyc1, Uqcrh, Cox5b,* and *Atp5g3*, were changed in the fenofibrate-treated cells (Table 1).

It has been shown that *Ndufa* genes play an essential role in the assembly pathway and function of NADH dehydrogenase (Complex I) in mammals [19]. Fenofibrate induced high expression levels of *Ndufa5* and *Ndufa10*, and low expression levels of *Ndufa1* (Table 1). *Ndufa1* was shown to interact with the subunits encoded by mitochondrial DNA during the Complex I assembly process [20]. Two missense mutations (G8R and R37S) in *Ndufa1* have been identified in male patients presenting with neurological syndromes [21]. *Ndufa5* is involved in building up the electrochemical potential required to produce ATP. To date, no mutations in *Ndufa5* are reported in association with any diseases. Nevertheless, low expression levels are found in the brain regions of those affected by autism [22, [23]. Mutations in *Ndufa10* lead to lowering holo-complex I levels with the accumulation of complex I subcomplexes, which indicates a disturbance in the assembly and/or stability of complex I [24]. The *Ndufb7* gene is classified in the hydrophobic group with NADH-binding and oxidizing properties [19, [25]. To a point, *Ndufa1, Ndufa5, Ndufa10*, and *Ndufb7* genes are likely to contribute a significant impact to PPARα response. However, function of the above 4 genes in the regulation of mitochondrial energy production and cancer development needs further investigation.

Additionally, fenofibrate up-regulated the expression of *Sdhd, Cyc1*, and *Atp5g3* genes. Succinate dehydrogenase (SDH) or Complex II is part of both the Krebs cycle and the electron transport chain [26]. The SDHD protein is the membrane-anchoring protein that contains one heme and is essential for ubiquinone binding. The Mitochondrial *Sdhd* gene is required for early embryogenesis [27]. Mutations in the *Sdhd* gene are associated with paraganglioma [28]. The cytochrome c1 (CYC1) protein, a subunit of respiratory chain complex III, directly interacts with cytochrome c and mediates electron transport from cytochrome b to cytochrome c during oxidative phosphorylation [29]. The *Cyc1* gene plays an important role in the development of nasopharyngeal carcinoma and osteosarcoma [30, [31]. The mitochondrial ATP synthase, subunit c, isoform 3 gene (*Atp5g3*) encodes subunit 9, which catalyzes ATP synthesis during oxidative phosphorylation in mitochondria. A recent report shows that the expression level of *Atp5g3* is reduced in autism patients [32]. In the present study, the up-regulated expressions of the *Sdhd, Cyc1*, and *Atp5g3* genes are consistent with metabolic reprogramming, which might switch the Warburg effect to oxidative phosphorylation with slowing energy production rate and inhibiting cancer cells growth in fenofibrate-treated cells. Recently, we have demonstrated that fenofibrate inhibits the invasion and migration of CAL 27 oral cancer cells through the AMPK and NF-κB signaling pathway [15].

*Cox5b* is an extra-membrane subunit located at the matrix side of the complex IV that facilitates the oxidation of cytochrome c by O_2_. *Cox5b* has been shown to play a role in suppressing ROS production [33, [34] and provide a binding site for protein kinase A [35]. *Uqcrh* is a component of the ubiquinol-cytochrome c reductase complex (complex III), which catalyzes electron transfer from succinate and nicotinamide adenine dinucleotide-linked dehydrogenases to cytochrome c [36]. *Uqcrh* is a downstream target gene of PGC-1α (Peroxisome proliferator-activated receptor gamma coactivator 1 alpha). The down-regulation of the *Uqcrh* gene suggests the decrease in mitochondrial oxidative phosphorylation activity, which results in reducing ROS accumulation and creating an antioxidant feedback [37]. Therefore, we suggest that the low expressions of *Cox5b* and *Uqcrh* in fenofibrate-treated cells are likely to contribute a significant impact to regulating ROS production involved in oral cancer development. Further investigation is needed to elucidate the association between ROS production and PPARα signaling.

## 5. Conclusions

The protein levels of PPARα were negatively associated with oral tumor progression. Activation of PPARα inhibited oral cancer cell migration. The molecular mechanism may be linked to modulate the expression of genes involved in mitochondrial energy metabolism.
